# Nanoparticles for “two color” ^19^F magnetic resonance imaging: Towards combined imaging of biodistribution and degradation

**DOI:** 10.1016/j.jcis.2019.12.083

**Published:** 2019-12-18

**Authors:** Olga Koshkina, Paul B. White, Alexander H.J. Staal, Ralf Schweins, Edyta Swider, Ilaria Tirotta, Paul Tinnemans, Remco Fokkink, Andor Veltien, N. Koen van Riessen, Ernst R.H. van Eck, Arend Heerschap, Pierangelo Metrangolo, Francesca Baldelli Bombelli, Mangala Srinivas

**Affiliations:** a Department of Tumor Immunology, Radboud Institute for Molecular Life Sciences, Radboud University Medical Center, Geert Grooteplein Zuid 26/28, 6525 GA Nijmegen, the Netherlands; b Institute for Molecules and Materials, Radboud University, Heyendaalseweg 135, 6525 AJ Nijmegen, the Netherlands; c Institut Laue – Langevin, DS/LSS, 71 Avenue des Martyrs, 38000 Grenoble; d Laboratory of Supramolecular and Bio-Nanomaterials (SupraBioNanoLab), Department of Chemistry, Materials, and Chemical Engineering “Giulio Natta ”, Politecnico di Milano, Via Luigi Mancinelli 7, 20131 Milan, Italy; e Department of Agrotechnology and Food Sciences, Physical Chemistry and Soft Matter, Wageningen University, 6708 WE Wageningen, Netherlands; f Department of Radiology and Nuclear Medicine, Radboud University Medical Center, Geert Grooteplein Zuid 10, 6525 GA Nijmegen, the Netherlands

**Keywords:** Fractal nanoparticles, Fluorocarbons, PLGA, SANS, ^19^F MRI, Degradation

## Abstract

The use of polymeric nanoparticles (NPs) as therapeutics has been steadily increasing over past decades. *In vivo* imaging of NPs is necessary to advance the therapeutic performance. ^19^F Magnetic Resonance Imaging (^19^F MRI) offers multiple advantages for *in vivo* imaging. However, design of a probe for both biodistribution and degradation has not been realized yet. We developed polymeric NPs loaded with two fluorocarbons as promising imaging tools to monitor NP biodistribution and degradation by ^19^F MRI.

These 200 nm NPs consist of poly(lactic-*co*-glycolic acid) (PLGA) loaded with perfluoro-15-crown-5 ether (PFCE) and PERFECTA. PERFECTA/PFCE-PLGA NPs have a fractal sphere structure, in which both fluorocarbons are distributed in the polymeric matrix of the fractal building blocks, which differs from PFCE-PLGA NPs and is unique for fluorocarbon-loaded colloids. This structure leads to changes of magnetic resonance properties of both fluorocarbons after hydrolysis of NPs.

PERFECTA/PFCE-PLGA NPs are colloidally stable in serum and biocompatible. Both fluorocarbons show a single resonance in ^19^F MRI that can be imaged separately using different excitation pulses. In the future, these findings may be used for biodistribution and degradation studies of NPs by ^19^F MRI *in vivo* using “two color” labeling leading to improvement of drug delivery agents.

## 1 Introduction

Polymeric nanotherapeutics have been developed for several decades, as they promise to improve the delivery of drugs and reduce side effects, and thus can have a strong impact on a treatment of various diseases [1–3]. Nevertheless, current drug delivery systems are still hampered by several drawbacks, to mention a few: low accumulation at the target site, insufficient control of drug release, and rapid blood clearance [3,4]. Monitoring biodistribution and degradation of NPs *in vivo* is essential to overcome these drawbacks, which has led to increased interest in theranostic NPs that additionally encapsulate an imaging agent [5–7]. Labeling NPs for monitoring both biodistribution and degradation *in vivo* without being limited by penetration depth remains a challenge to this day. Here, we developed polymeric NPs labeled with two different fluorocarbons, which hold a huge potential to act as a biodistribution and degradation probe with quantitative ^19^F MRI, due to their structural properties.

“Hot spot” fluorine MRI, which uses the magnetic moment of ^19^F nucleus, in addition to proton, has gained a lot of attention for clinical imaging over past years [8,9]. The signal from the ^19^F nucleus is directly detected, in contrast to SPIO or gadolinium-based labels, which are indirectly observed by modulation of the proton signal. The ^19^F nucleus has a unique combination of characteristics for imaging: (1) it has no endogenous background signal [10], (2) it displays a broad chemical shift for simultaneous “multi-color” *in vivo* tracking of different compounds [11–14], and, finally, (3) the obtained signal is linearly correlated to the amount of fluorine atoms, enabling quantitative imaging [15]. These properties make ^19^F MRI suitable for *in vivo* tracking of the biodistribution of labeled cell therapies [15–19], drug carriers [20,21], and molecular imaging agents [22]. None of the other imaging modalities combine all of these features.

Typically, liquid perfluorocarbons are used as a label for ^19^F MRI [8,10]. Perfluorocarbons contain high amount of fluorine nuclei and display balanced relaxation times that yield high signal intensity. Perfluoro-15-crown-5 ether (PFCE) is an ideal candidate for imaging, as it contains 20 chemically-equivalent fluorine nuclei that result in a single resonance signal, avoiding chemical shift artifacts. More recently, a new single-resonance probe PERFECTA, which is a crystalline solid at physiological temperature and contains 36 equivalent fluorine nuclei, was introduced [23].

Despite advantages for imaging, stabilizing perfluorocarbons in an aqueous solution is challenging, due to their hydrophobic and lipophobic character [24]. Typical imaging probes for ^19^F MRI are nanoemulsions stabilized with phospholipids or core-shell capsules with a polymeric shell and a core of a liquid perfluorocarbon [8,10,25,26]. Recently, we described PFCE-poly(lactic-*co*-glycolic acid) (PLGA) NPs that have a fractal multicore structure which contains multiple PFCE domains within one polymeric particle [27–29]. These NPs have been successfully used as cell tracking agents with ^19^F MRI. Moreover, they are cleared several times faster *in vivo* compared to core-shell systems, which is an advantage for biomedical applications [30].

For drug delivery applications tracking not only biodistribution, but also degradation of NPs is important. There are only few potential ^19^F labels, which could be used for *in vivo* tracking of NP break-down. However, these labels are unsuitable to monitor the biodistribution of the intact label [31,32]. In fact, there are major differences between tracking agents for NP biodistribution and NP degradation. Probes for NP degradation have an extremely short transverse relaxation time T_2_ to completely suppress the signal of ^19^F nuclei in the intact material. The suppression of the signal can be achieved either by sterically blocking fluorine atoms [31] or by coupling paramagnetic metal chelates, such as gadolinium [32,33], with the fluorinated compound. Thus, the ^19^F MRI signal can be detected only after degradation of the nanomaterial, hampering *in vivo* tracking of the intact nanomaterial. Simultaneous tracking of biodistribution and degradation of NPs have not been realized so far.

We hypothesized that combining two fluorinated molecules within the same particle could enable design of a probe for both biodistribution and degradation monitoring, given that both fluorocarbons separate from each other upon degradation. Due to miscibility gap between the perfluorocarbon and aqueous phase, it is unlikely that the two perfluorocarbons that are encapsulated within a core-shell structure will diffuse apart after degradation of the shell. However, for fractal structure this behavior was not clear. Therefore, the goals of this work were to develop nanoparticles loaded with two different fluorocarbons to study their colloidal properties, with focus on the structure and fluorocarbon interface, and, finally, to investigate their magnetic resonance properties upon hydrolysis, providing a proof of principle for our concept.

We decided to focus on PFCE and PERFECTA [23] as both molecules display a single resonance signal that would be advantageous for imaging allowing the use of common sequences. As a polymer for encapsulation we used PLGA, as this is a biodegradable and biocompatible polymer, which finds a broad use in drug delivery and controlled release applications [34]. We have developed the production method and performed intensive structural characterization. These data showed that nanoparticles have structural properties that potentially enable tracking of biodistribution and degradation, confirming the hypothesis. Moreover, nanoparticles were shown to be biocompatible and could be imaged with ^19^F MRI using a conventional sequence within a short time demonstrating that they will be suitable for *in vivo* imaging.

## 2 Materials and methods

### 2.1 Materials

Water was purified with a purification system from Merck Millipore. The following chemicals were used as received: poly(d,l-lactide-*co*-glycolide) (PLGA) Resomer^®^ RG 502H, lactide:glycolide molar ratio 50:50 (Evonik Industries AG, Essen, Germany). 1,1,1,3,3,3-hexafluoro-propan-2-ol, ≥99% (HFIP) and poly(vinyl alcohol) (PVA, M_w_ 9000–10000 g/mol, 80% hydrolyzed, Prod. Nr. 360627, MKBF2590V) from Sigma Aldrich, Germany; dichloromethane, Ph. Eur. (DCM) (Merck, Darmstadt, Germany). Perfluoro-15-crown-5-ether (PFCE), 99%, was purchased from Exfluor, Round Rock USA. PERFECTA was synthesized as described previously [23]. For cell culture X-VIVO 15 medium (Lonza, Belgium) supplemented with 2% human serum was used. Phosphate-buffer saline (PBS) (Braun, Germany) was used to resuspend NPs for cell experiments and for cell washing.

#### 2.1.1 Preparation of the NPs

For the synthesis of the NPs loaded with PERFECTA and PFCE, PERFECTA (100 mg, 0.1 mmol), PFCE (0.9 mL, 1.6 g, 2.8 mmol), and PLGA (100 mg, M_w_ = 7–17 kDa) were dissolved in HFIP (3 mL). The resulting solution was rapidly added with a glass pipette to an aqueous solution of poly(vinyl alcohol) (25.5 g, 1.96 wt-%) in a round bottom flask while sonication was started. The entire mixture was sonicated in ice-water bath for 3 min at an amplitude of 40% (3.2 mm microtip, Branson digital sonifier s250). After sonication the resulting emulsion was transferred into a beaker and stirred overnight at room temperature to evaporate the solvent (the evaporation of solvent seemed faster in a beaker).

The particles were isolated by centrifugation at 16,087*g* (11000 rpm) for 35 min in 50 mL centrifugation tubes and resuspended in 25 g of water followed by another centrifugation step. After washing, particles were resuspended in 4 mL of water, frozen with liquid N_2_ and freeze-dried. The resulting product was a white powder with a yield of typically 100 mg. Typical encapsulation yield of PERFECTA was 8.2 ± 1.7 wt-% and of PFCE 15 ± 4.7 wt-% (n = 3)

PFCE-PLGA NPs were prepared using a miniemulsion formulation method as described previously [27,28]: PLGA (100 mg) was dissolved in 3 mL dichloromethane followed by addition of perfluoro-15-crown-5 ether (0.9 mL). The resulting double phase liquid was rapidly mixed by pipetting it up and down and added with a glass pipette to solution of poly(vinyl alcohol) (25.5 g, 1.96 wt-%) in a round bottom flask while sonication was started. As PFCE does not mix with PLGA in DCM, PFCE and PLGA/DCM were pipetted up and down during the addition to ensure that both phases are added simultaneously. The entire mixture was sonicated in ice-water bath for 3 min at an amplitude of 40% (3.2 mm microtip, Branson digital sonifier s250).

After sonication, dichloromethane was evaporated at 4 °C overnight under stirring to achieve solidification of the particles. The particles were isolated by centrifugation at 16,087*g* for 35 min in 50 mL centrifugation tubes and resuspended in 25 g of water. The washing step was repeated three times with sonication in ultrasonic bath after second washing (sonication bath, Diagenode Bioruptor). After washing, particles were resuspended in 4 mL of water, frozen with liquid N_2_ and freeze-dried. The freeze-dried particles were collected as a white powder with a typical yield of 100 mg.

### 2.2 Characterization methods

*CryoSEM* was measured at JEOL 6330 Cryo Field Emission Scanning Electron Microscope (FESEM). The samples (8 μL) were pipetted in 2 rivets, which were then placed together. Next, the samples were frozen in liquid nitrogen slush and placed in an Oxford Alto 2500 cryo station with a cryo-transfer device. There the top rivet was broken and the sample was heated to −95 °C for 5 min, followed by a coating of 60/40 Au/Pd and transfer to the Cryo-SEM.

*Multiangle DLS and Static LS* were measured at ALV compact goniometer system equipped with ALV7004 correlator, ALV/LSE-5004 Goniometer, ALV / Dual High QE APD detector unit with fiber splitting device a set-up of 2 off detection system and a Uniphase Model 1145P He-Ne Laser. The laser wavelength and power were 632.8 nm and 22 mW, respectively. The temperature was controlled by a Julabo CF41 thermostatic bath. Water for dilutions was filtered with 0.45 μm hydrophilic filters. The concentration of samples was 0.01 mg/mL. For size determination under physiological conditions, NPs were added to RPMI or RPMI with 5 vol-% FBS, which were filtered through Millex-LCR filter, 0.45 μm pore size. The measurements were repeated at least three times.

DLS was measured at scattering angles θ = 30–150° in 10° steps. Data analysis was done with HDRC software, which was kindly provided by Prof. Manfred Schmidt, University of Mainz, Germany. The apparent hydrodynamic radii at different angles were obtained from a biexponential fitting of autocorrelation function, as it provides a good approximation of the radius for particles with polydisperse, monomodal size distribution. The absolute inverse z-averages of hydrodynamic radii were obtained by extrapolating the apparent radii q → 0. The second cumulant values μ_2_ were obtained from cumulant fitting at θ = 90°

Size determination under physiological conditions was done using the multicomponent analysis with HDRC software. Here, the autocorrelation function of nanoparticle-protein mixture was spited in a triexponential fit function for FBS (known component) and biexponential fit function for NPs (unknown component) [35]. The fitting coefficients of FBS were previously obtained from triexponential fitting of serum without NPs.

SLS for determination of radius of gyration *R_g_* was measured at θ = 30–60° in 3° steps. This measurement range was selected, as it is needed for Guinier analysis (q × R_g_ ≤ 1).

The radius of gyration was determined from the angular dependence of the Rayleigh ratio according to the Guinier equation. The Rayleigh ratio *R_θ_* was obtained as follows:

$$R_{\theta }=\frac{I{\left(\theta \right)}_{\text{sample}}-I{\left(\theta \right)}_{\text{water}}}{I{\left(\theta \right)}_{\text{toluene}}}\times R_{\text{toluene}}\times \frac{n_{\text{water}}^{2}}{n_{\text{toluene}}^{2}}$$

With a refractive index of solvent n_water_ = 1.333, refractive index of reference *n_toluene_* = 1.494 and Rayleigh ratio of toluene *R_toluene_* = 1. 02 × 10^−3^ m^−1^ calculated as described by Wu [36].

*Nuclear magnetic resonance spectroscopy* was measured on Bruker Avance III 400 MHz spectrometer equipped with BBFO probe at 298 K (quantitative measurements and NOESY), or at Bruker Avance III 500 MHz spectrometer equipped with Prodigy BBO cryoprobe (relaxation measurements). Particles (typically 5–8 mg) were dissolved in 600 μL D_2_O. A 90° pulse was calibrated prior measurements.

2D Nuclear Overhauser Enhancement Spectroscopy (NOESY) was performed using a spectral width spanning from (−102) to (−62) ppm for ^19^F (377 MHz) using 2048 (F2) × 128 (F1) points. 96 scans per increment were acquired using an interscan relaxation delay of 2.0 s and a mixing time for dipole-dipole relaxation of 250 ms.

Longitudinal relaxation times T_1_ were measured using an inverse recovery sequence and transverse relaxation times T_2_ using the CPMG sequence. Relaxation times were measured with spectral width spanning from typically (−101) to (−62) ppm. At least 12 data points were acquired, which were then used for data fitting. An interscan delay was set to 5 × T_1_, which was estimated using a 1D version of the inversion recovery sequence and noting the null crossing point.

Quantitative ^19^F NMR was for determination of fluorocarbon content was measured in D_2_O as a solvent using known amount of NPs with trifluoroacetic acid (typically 1 μL) or sodium triflate (typically 1 mg) as a reference for quantification. An interscan relaxation delay was 20 s with TFA or 30 s with sodium triflate. The number of scans was between 8 and 32 depending on concentration of fluorinated compound.

Data evaluation was done with MestreNova 10.0 from Mestrelab (quantification, relaxation times) or with TopSpin 3.5 from Bruker (NOESY).

*^19^F and ^13^C solid state NMR* were measured on a Varian 400 MHz solid state NMR system using a 3.2 mm T3 design probe resonant for ^19^F at 376.272 MHz and ^13^C at 100.569 MHz. Magic angle spinning was employed at 16 kHz with 90° pulses of 2 μs for ^19^F and 3.75 μs for ^13^C. Ramped cross polarization from ^19^F to ^13^C was set-up on PTFE (Teflon). The Hartmann-Hahn matching condition was achieved with 78 kHz rf-field strength on ^19^F and 62 kHz on ^13^C with the difference the MAS frequency. Spinal decoupling with a pulse length of 7.2 μs and 7°phase was used to decouple ^19^F from ^13^C.

*XRD* was measured on a Panalytical Empyrean in reflection mode with fine-focus sealed tube, and PIXcel3D detector, using Cu Kα radiation. The scan range was from 2 to 50° 2-theta, with a step size of 0.013°.

*Small Angle Neutron Scattering (SANS)* was measured at D11 small angle diffractometer at ILL Grenoble and at Sans2d at ISIS spallation neutron source UK [37]. The description of settings of sans2d diffractometer is given in detail in supplementary information.

*SANS* measurements were done on D11 refractometer using 3 sample-detector distances of 1.5 m, 8 m and 39 m, with corresponding collimation distances of 8 m, 8 m and 40.5 m. The wave-length used was 6 Å, with a FWHM of 9%. Cylindrical Hellma cells were placed in a thermostated sample changer, the temperature was kept constant at 25 °C. A neutron beam of 13 mm in diameter was employed.

Scattering intensities were recorded using a two-dimensional position-sensitive ^3^He detector with a pixel size of 7.5 × 7.5 mm^2^ and an active area of 96 × 96 cm^2^. A dark current was measured and subtracted from all data. Solvents were measured and subtracted, too, taking into account the transmission values of all samples and solvents as obtained by a dedicated measurement. The data were first corrected pixel by pixel and in a second step radially regrouped in order to obtain a one-dimensional scattering curve in the form Intensity vs. Q. Data were put on an absolute scale making use of the secondary calibration standard H_2_O (cross-calibrated against h/d polymer blends), which has been measured in a 1 mm Hellma cell. The differential scattering cross section for H_2_O with a thickness of 1 mm has been determined to 0.983 1/cm for D11 at 6 Å neutron wavelength and 25 °C. Data were reduced using ILL’s standard software package LAMP [38,39].

Data analysis was done using the NIST SANS package, version 7.50, for Igor Pro (Wavemetrics) [40].

#### 2.2.1 Cell culture and labeling

Peripheral blood mononuclear cells (PBMCs) were isolated from buffy coats of healthy individuals after informed consent, using Ficoll density centrifugation (Lymphoprep, STEMCELL Technologies, Vancouver, Canada). Adherent monocytes were cultured in X-VIVO 15 medium supplemented with 2% human serum, and in the presence of interleukin-4 (300 U/mL) and granulocytemonocyte colony stimulating factor (450 U/mL) to obtain immature dendritic cells (DCs). Day 3 cells were harvested and labeled with NPs (resuspended in PBS right before the labeling) at concentration 2 mg of NPs/ 1×10^6^ cells.

#### 2.2.2 Cell viability

To investigate the influence of labelling on cell viability we performed an MTT assay. For this, 1×10^6^ DCs were incubated in the presence of NPs for 72 h. Here, both PFCE-PLGA-NPs and PERFECTA/PFCE-PLGA-NPs samples were tested, including positive and negative control. After incubation, the excess of the label was removed by gentle washing with PBS. Next, cells were collected and placed in a 96-well flat-bottom plate, washed two time with PBS (100 μL/well) with centrifugation of 2 min between each wash. Next, 60 μL X-VIVO medium with 10 μL MTT (3-(4,5-dimethylthia zol-2-yl)-2,5-diphenyltetrazolium bromide) (concentration 4 mg/mL) was added to each well, followed by 1 h incubation at 37 °C. After incubation, the plate was centrifuged for 2 min and 100 μL of lysis buffer (isopropanol, 10% SDS, 2 N HCL, deionized water) was added to each well, and the plate was incubated for 15 min in dark at room temperature. Before the measurement, samples were resuspended to remove any precipitate of crystals. The plate was measured with iMark™ microplate reader (Bio-Rad, the Netherlands) at 595 nm. Cell not loaded with NPs were used as negative control and cells treated with DMSO were used as a positive control.

#### 2.2.3 ^19^F magnetic resonance imaging

^19^F MRI was performed on a pre-clinical 11.7 T MRI scanner (Biospec 117/16, 500 MHz, Bruker, Ettlingen, Germany). PERFECTA/PFCE-PLGA NPs were homogeneously dissolved at a concentration of 10 mg/mL, the PFCE-only NPs and PERFECTA emulsion were diluted to contain a corresponding amount of fluorine atoms. The phantoms were imaged with a selective excitation 3D RARE-sequence with the following parameters: TR = 1500 ms, TE = 6.62 ms, RARE-factor 44, matrix size 64x44, FOV 32 × 22 mm, 16 slices with a thickness of 2 mm, 5 averages for a scan time of 2 min. To selectively excite, a transmission bandwidth of 1000 Hz was used, PERFECTA was excited at 470,753,521 Hz and PFCE at 470,744,387 Hz. To determine concentration limit of the probe we scanned the phantoms at various voxel sizes. The keep clinical applicability and *in vivo* relevance in mind, the scan time and reference voxel size were chosen accordingly. For scan time we used 8 min per excitation frequency to make a total of 16 min for both PFCs. For voxel size we aimed for 1 × 1 × 2 mm, as this is a sufficient resolution in our experience. Detection limit was determined to be a voxel size of 0.33 × 0.33 × 2 mm within the 8 min, this is a voxel size 10-fold smaller than the eventual *in vivo* voxel size. Therefore, a 1/10th concentration of the phantom concentration is the concentration limit.

## 3 Results and discussion

### 3.1 Synthesis and characterization of the NPs

NPs loaded with liquid fluorocarbons can be typically produced with a miniemulsion formulation method using non-fluorinated solvents [28]. PERFECTA, however, is a solid, which is poorly soluble in most non-fluorinated organic solvents. Our usual miniemulsion formulation method for PFCE-PLGA-NPs with dichloromethane as a solvent did not yield a sufficient encapsulation of PERFECTA. Therefore, we used 1,1,1,3,3,3-hexafluoro-pro pan-2-ol (HFIP), which is suitable to dissolve PLGA, PERFECTA, and PFCE. The solution of PLGA, PERFECTA, and PFCE in HFIP was added to aqueous solution of poly(vinyl alcohol) (PVA) as a nonionic surfactant and emulsified using sonication to provide the energy that is required for encapsulation of fluorocarbons [28]. As HFIP is miscible with water, the final process is a combination of miniemulsion and nanoprecipitation technique.

**Fig. 1:**
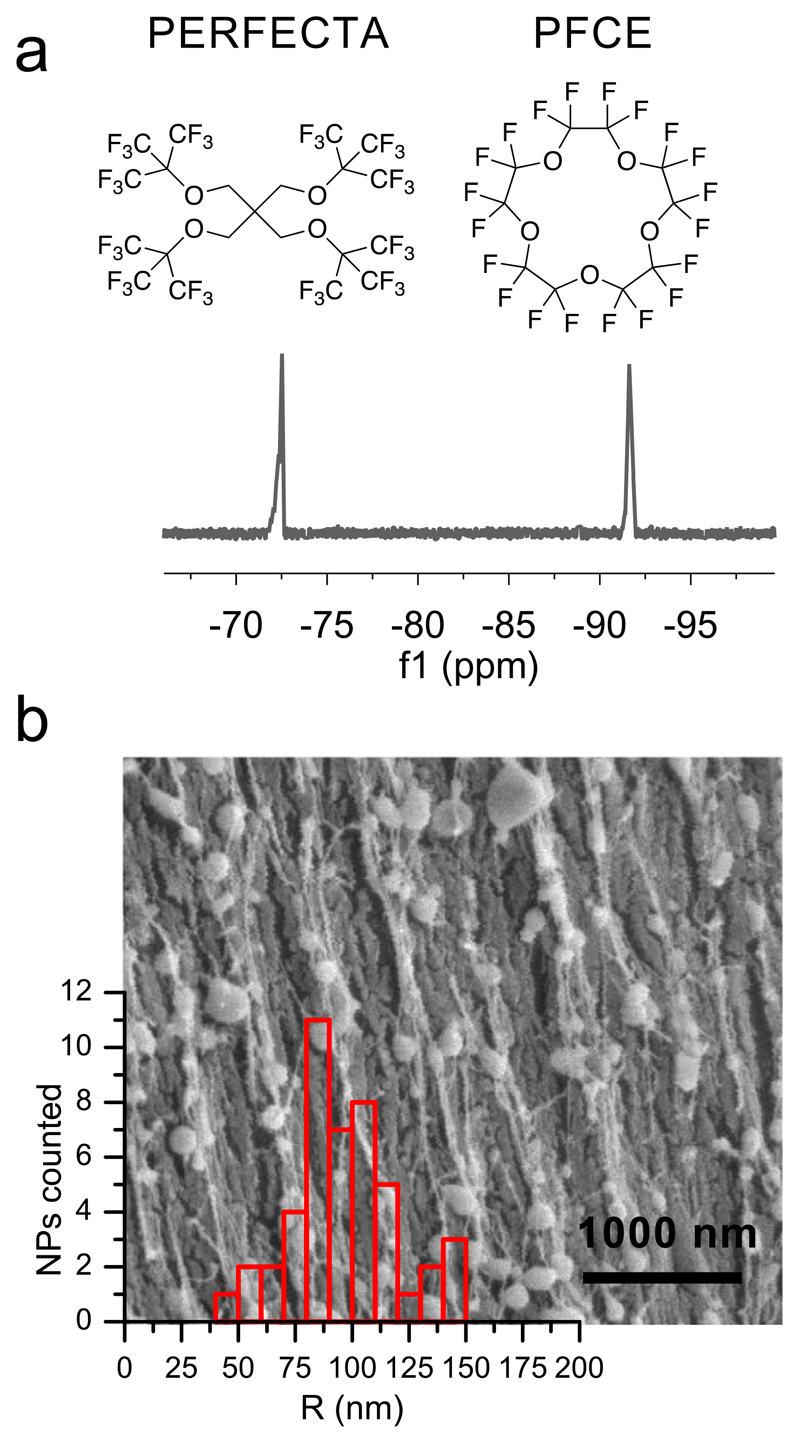
Characterization of PERFECTA/PFCE-PLGA NPs. (a) ^19^F NMR demonstrates that both PFCE and PERFECTA were encapsulated in the NPs (471 MHz in D_2_O). (b) CryoSEM shows that the majority of NPs have a mean radius of 96 ± 23 nm (47 NPs measured; c_NP_ = 10 mg/mL in water). However, small numbers of larger and smaller particles could be detected. Scale bar 1000 nm.

^19^F NMR spectroscopy on purified, freeze-dried NPs after redispersion in water demonstrated that both fluorocarbons were encapsulated in the NPs (Fig. 1a). The content of PERFECTA in the final NPs was 8.7 wt-% and of PFCE 10 wt-%, with some batch-to-batch variation (compare materials and methods), as shown by NMR spectroscopy. Cryogenic Scanning Electron Microscopy (cryo-SEM, Fig. 1b) demonstrated that the mean radius of the NPs was 96 ± 23 nm, indicating a certain polydispersity which is typical of PLGA NPs [34]. The average hydrodynamic radius of NPs was 164 ± 7 nm (second cumulant μ_2_ (90°) = 0.2) as determined by multi-angle Dynamic Light Scattering (scattering angle *θ* = 30–150°, compare Fig. S1). The radius of gyration *R_g_* was 167 nm, as revealed by Guinier analysis of Static Light Scattering (SLS, *θ* = 30–60°, Fig. S1). The difference between multi-angle DLS and cryoSEM results from higher sensitivity of light scattering to bigger particles at small scattering angles [41]. Overall, the size distribution was comparable to PFCE-PLGA-NPs [28]. The zeta-potential of NPs was –5 mV ± 0.4 mV (in 5 mM sodium chloride), which is typical for colloids that are sterically stabilized with a non-ionic surfactant, such as PVA.

While this method works well for obtaining PLGA NPs only loaded with PFCE, it was not possible to obtain PLGA-NPs loaded only with PERFECTA that had a narrow size distribution (compare supplementary information, section 1). Thus, the presence of the two fluorocarbons seem to be necessary to formulate PVA-stabilized PLGA NPs loaded with PERFECTA.

### 3.2 Structural characterization of the NPs

For biomedical applications, physicochemical properties of NPs, in particular the internal structure of NPs is important, as it can affect drug loading and/or the mechanism of degradation. Therefore, we performed an in-depth characterization of PERFECTA/PFCE-PLGA-NPs with different analytical techniques, to determine the arrangement of fluorocarbons inside the polymeric matrix.

**Fig. 2:**
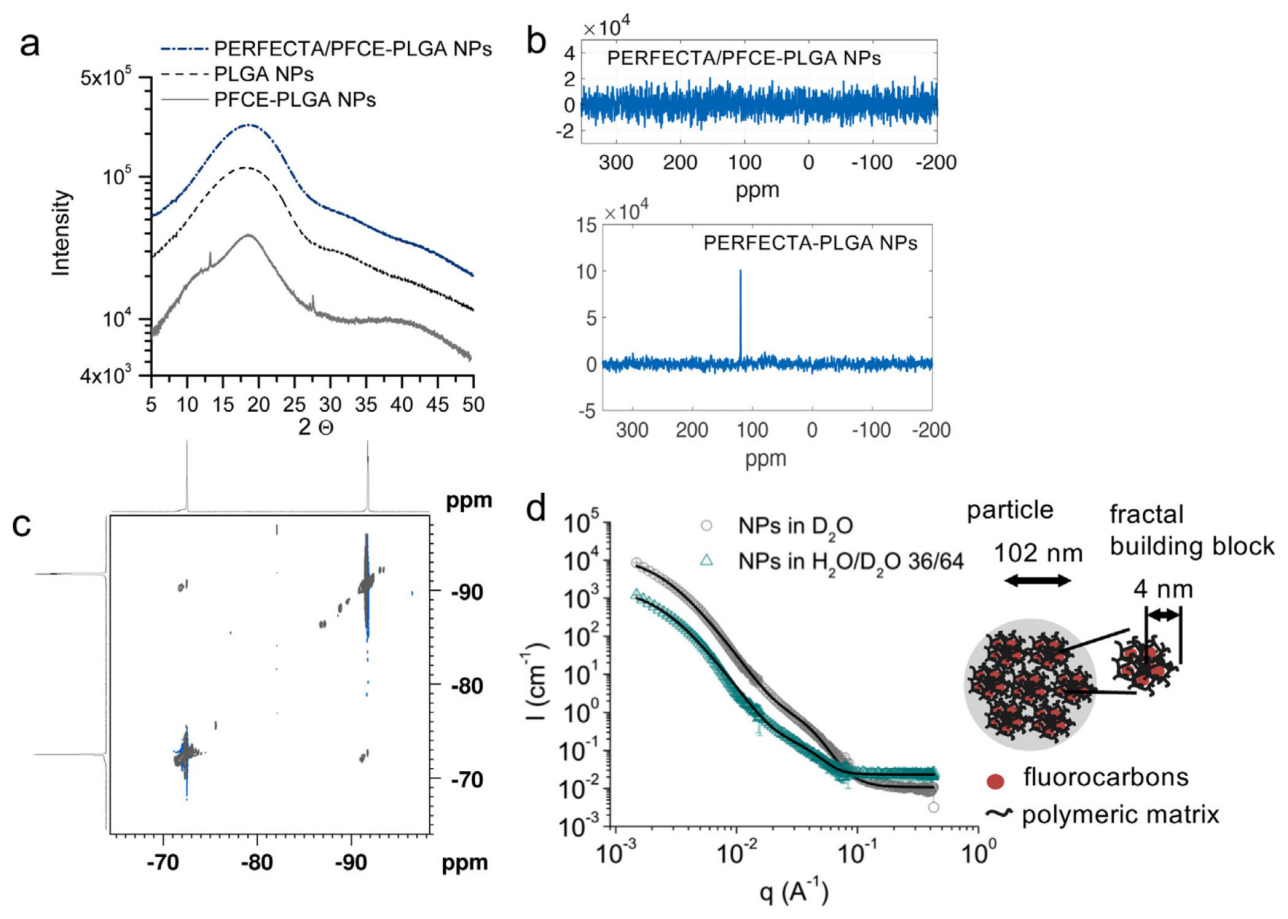
(a) Powder X ray diffraction of PFCE/PERFECTA-PLGA-NPs compared to PFCE-PLGA-NPs and PLGA-NPs. No crystalline peaks could be detected, indicating that PERFECTA is mixed with PFCE and polymeric matrix. (b) ^13^C solid state NMR spectra obtained by cross-polarization from ^19^F. (c) In ^19^F-^19^F NOESY measurements PFCE (−92 ppm) and PERFECTA (−73 ppm) display NOE effect (cross-peak between both compounds), demonstrating that PFCE and PERFECTA are close to each other. 376.5 MHz, in D_2_O. (d) SANS scattering patterns of PERFECTA/PFCE-PLGA-NPs in D_2_O and H_2_O/D_2_O 36/64 (match conditions for PFCE) with simultaneous fit of the particle form factor with a fractal sphere model. SANS reveals that PERFECTA/PFCE-PLGA-NPs have a fractal structure with spherical building blocks that contain PFCE, PERFECTA, and polymer. c (NP) = 10 mg/mL.

As pure PERFECTA is a crystalline solid at room or physiological temperature [23], we first measured powder X-ray diffraction (PXRD) to examine the crystallinity. PXRD patterns of PERFECTA/PFCE-PLGA-NPs were similar to PXRD patterns of PFCE-PLGA-NPs suggesting an amorphous structure of the NPs (Fig. 2a). As no crystalline peaks typical for PERFECTA [8] were detected, PXRD may indicates that PERFECTA loses its crystallinity upon encapsulation in PLGA matrix. Furthermore, cross polarization measurements between ^19^F and ^13^C nuclei by solid state NMR spectroscopy did not show any cross-polarization signal between the fluorocarbons (Fig. 2b, upper spectrum, corresponding ^13^C-spectra Fig. S2). As a control for these measurements we used PERFECTA-PLGA-NPs (compare section 1 in SI for synthesis and characterization). In contrast to PERFECTA/PFCE-PLGA-NPs, PERFECTA-PLGA-NPs displayed cross polarization between ^19^F and quaternary ^13^C nucleus of PERFECTA-molecule (Fig. 2b, lower spectrum). The absence of cross polarization signal in PERFECTA/PFCE-PLGA-NPs indicates that PERFECTA molecules have higher mobility within the polymeric matrix compared to PERFECTA-PLGA-NPs. Thus, PERFECTA molecules in PERFECTA/PFCE-PLGA-NPs show a liquid-like behavior, indicating that PERFECTA is in different environment and possibly close to PFCE.

To further study the interactions between both fluorocarbons in PERFECTA/PFCE-PLGA-NPs, we measured ^19^F-^19^F Nuclear Over-hauser Enhancement Spectroscopy (NOESY) on NPs in solution. NOESY detects cross-space magnetization transfer between different nuclei, when the distance between these nuclei is smaller than 6 Å. An NOE was observed between ^19^F nuclei of PFCE and PERFECTA, demonstrating that PFCE and PERFECTA molecules must be close to each other (Fig. 2c). This technique showed that F atoms of the molecules are close within the NPs, but not being a quantitative measurement, it is not possible to evaluate if all atoms or just some of them interact and to which extent.

Finally, we performed Small Angle Neutron Scattering (SANS) experiments at different solvent contrasts (100% deuterium oxide, water/deuterium oxide ratio 36/64 and 61/39 (*v:v*)) to describe the local structure of PERFECTA/PFCE-PLGA-NPs. In 100% deuterium oxide, all components of the NPs contribute to the scattering contrast. At a ratio of 36/64 (*v:v*) PFCE is matched and it does not contribute to the scattering (Fig. 2d, Table S2, Fig. S4 for results from a different beamline). For data analysis we performed model fitting of the absolute scattering intensity- the particle form factor, using a simultaneous fitting approach to reduce the number of fitted parameters.

Fluorocarbon-loaded colloids typically have a core-shell structure [8]. However, we have previously shown by SANS analysis that PFCE-PLGA-NPs have a fractal multi-core structure that consists of a fractal assembly of core-shell building blocks (NIST-model FractalPolyCore) [27]. Nevertheless, the form factor of PERFECTA/PFCE-PLGA-NPs could not be described by either of these models. In contrast, a fractal sphere model developed by Teixeira provided a good fit of the scattering curves (Fig. 2d, Table 1) [42]. This model describes a NP as a fractal assembly of spherical building blocks formed of PFCE, PERFECTA, and PLGA. The final particles are composed of spherical fractal building blocks of 4 nm, and have a radius of gyration *R_g_* of 102 nm, as derived from the correlation length. A schematic representation of the interior structure of the PERFECTA/PFCE-PLGA-NPs is shown in Fig. 2 [42]. In PERFECTA/PFCE-PLGA-NPs, the encapsulated fluorocarbons are homogenously distributed between PLGA chains forming mixed spherical fractal building blocks. This arrangement is different from PFCE-PLGA-NPs, in which PFCE forms fractal building blocks composed of a fluorocarbon core surrounded by a PLGA shell (compare also Fig. S3 for differences between fractal sphere and fractal core-shell, NIST-models FractalPolySphere or FractalPolyCore). We expect that both fluorocarbons form very small clusters between polymeric chains. The resulting R_g_ of 102 nm is close to the value of the mean radius of 96 nm obtained from cryo-SEM analysis, confirming the results of model fitting.

Based on the content of PFCE and PERFECTA obtained from NMR spectroscopy, the expected scattering length density (SLD) of a fractal building block can be estimated to be 2.6 × 10^−6^ Å^−2^. In both solvents, the SLD obtained from the model fit is between the calculated SLD of particles and the SLD of solvent, indicating that the NPs might be hydrated with a solvent (Table 1).

NMR relaxation time measurements confirmed the structural difference between PERFECTA/PFCE-PLGA-NPs and PFCE-PLGA-NPs (Table 2). Both longitudinal (T_1_) and transverse (T_2_) relaxation times of PFCE were shorter in PERFECTA/PFCE-PLGA-NPs than in pure PFCE-PLGA-NPs. The effect is especially pronounced for T_2_ that is more sensitive to the environment of the nucleus than T_1_. The shortening of relaxation times is typical for molecules in confined environment, due to their lower mobility [43]. According to the SANS model, in PERFECTA/PFCE-PLGA-NPs, both fluorocarbons are distributed between the polymeric chains. In contrast, in PFCE-PLGA-NPs, PFCE forms several domains within the nanoparticle and has therefore, higher mobility. Additionally, the presence of PERFECTA can further contribute to the reduction of T_2_.

Usually, fluorocarbon-loaded colloids form core-shell structures with a core of liquid fluorocarbon, due to poor miscibility of fluorocarbons with both aqueous and organic emulsion phases. The only examples where the internal structure was different include PFCE-PLGA-NPs reported previously by our group [27] and multicore micelles formed by thermosensitive polymers with covalently-attached fluorocarbon and hydrocarbon side chains [44,45]. At last, the driving force for the formation of domains was still the tendency of fluorocarbons to segregate from hydrocarbon side chains. However, here the driving force for the formation of fractal structure seems to be different.

Possible explanation for the formation of such a fractal sphere structure composed of mixed PERFECTA, PFCE, and PLGA building blocks, in which all components are distributed homogenously, could be related to the use of HFIP as a solvent. In fact, HFIP allows dissolution of both fluorocarbons and PLGA and, being also miscible with water, might diffuse into the aqueous phase upon addition of organic HFIP solution to aqueous surfactant solution promoting the formation of PERFECTA/PFCE-PLGA building blocks arranged in a fractal spherical structure.

Both PERCECTA and PFCE are non-miscible with PLGA, and thus the formation of fractal structure seems not to be thermodynamically favored. Thus, kinetic effects, such as diffusion of solvent, are likely to play a role in the structure formation. Further effects could also influence the formation of the NPs. Particularly, we observed that the impurities in the surfactant, such as poly(propylene glycol) or shorter PVA chains, may generally affect the encapsulation of fluorocarbons, independent of the solvent or the method used.

### 3.3 ^19^F magnetic resonance properties alter after degradation of NPs

Finally, we wanted to test if both fluorocarbons can separate from each other upon the breakdown of the polymeric matrix. In the body, PLGA is usually metabolized to lactic and glycolic acid, which then enter into the tricarboxylic acid cycle [34]. After PLGA degradation, the surroundings of both fluorocarbons will change, leading to altered magnetic resonance properties. To study the behavior of both fluorocarbons after degradation, we hydrolyzed PERFECTA/PFCE-PLGA-NPs with aqueous sodium hydroxide solution. The solution of NPs turned from milky to transparent within few minutes after the addition of sodium hydroxide, indicating the success of hydrolysis. In ^1^H NMR spectra, signals from lactic and glycolic acid monomers could be detected (Fig. S5). In contrast, before the hydrolysis, no PLGA signals were observed in proton spectrum due to the reduced mobility of polymeric chains in the particles (Fig. S5). The choice of using alkaline conditions is related to the ^1^H NMR measurement after hydrolysis, for avoiding additional proton signals from water or organic acids in the same region as PLGA or PVA protons. Despite NPs would be hydrolyzed in acidic environment within the cell, acidic and alkaline hydrolysis are substitution reactions leading to the same final product.

To further investigate the effect of degradation on the ^19^F signal, we measured longitudinal and transverse relaxation times, T_1_ and T_2_, of both fluorocarbons before and after hydrolysis. Additionally, we compared the results with PFCE-PLGA-NPs (Table 2). After the hydrolysis of PERFECTA/PFCE-PLGA-NPs, T_2_ of both fluorocarbons increased, indicating that the fluorocarbons became more dynamic after the degradation of PLGA-matrix. In contrast, T_2_ decreased after degradation in PFCE-PLGA-NPs. Before hydrolysis PFCE-PLGA-NPs displayed longer transverse relaxation T_2_ compared to PERFECTA/PFCE-PLGA-NPs, with a difference of around 200 ms, which might be attributed to a different internal structure of both types of colloids. After hydrolysis, the difference between T_2_ of PFCE in PFCE-PLGA-NPs and PERFECTA/PFCE-NPs is only 43 ms. These changes in T_2_ may suggest that the surrounding environment of PFCE is similar after hydrolysis in both types of NPs.

After hydrolysis we could not observe any visible phase separation of fluorocarbons. In contrast, when a similar amount of PFCE was added to water, the phase separation could be observed by eye. DLS of hydrolyzed PERFECTA/PFCE-PLGA-NPs measured at a scattering angle of 173° showed the radius of 89 nm (PDI = 0.22), indicating that some colloidal structures were still present in the solution. However, the scattering intensity of this solution was lower than that of non-hydrolyzed NPs. The results were obtained without attenuation, while for measurements of intact particles of same concentration 36% attenuation were used (both set automatically by DLS machine). The size determination of hydrolyzed PFCE-PLGA-NPs was not possible due to high polydispersity of the obtained solution; the aggregates were around 200–300 nm. This behavior suggests that both fluorocarbons are still stabilized by the remaining surfactant after hydrolysis of PLGA matrix.

**Fig. 3:**
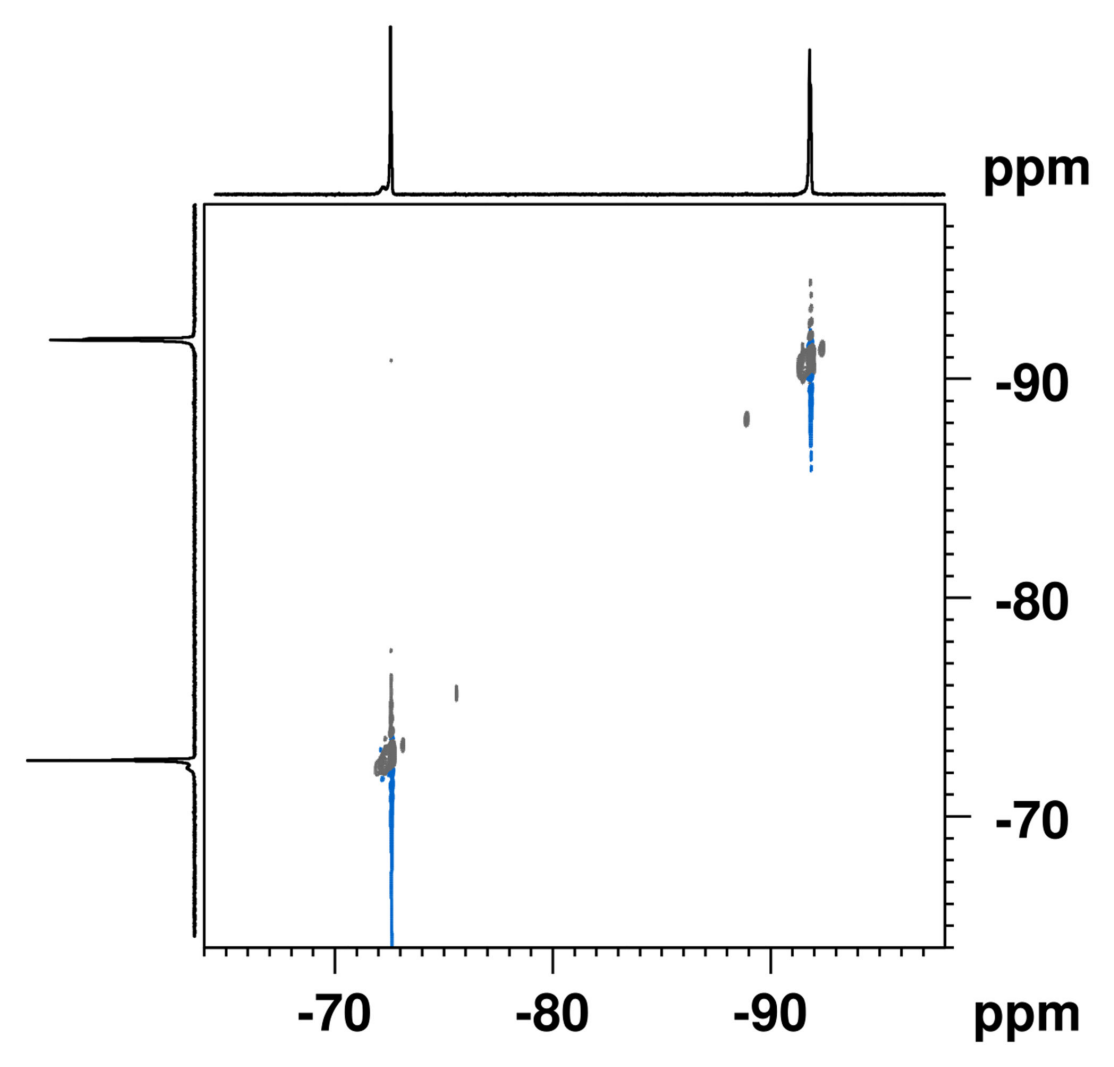
NOE-spectrum of PERFECTA/PFCE-PLGA-NPs after hydrolysis with sodium hydroxide. The absence of NOE-signal indicates the increased distance between both fluorocarbons after degradation of NPs. 400 MHz (^1^H), 376.5 MHz (^19^F), in D_2_O.

To further obtain information about the environment of both fluorocarbons after hydrolysis, we performed NOESY experiments. In contrast to intact particles, we could not detect sufficient NOE between PFCE and PERFECTA anymore (Fig. 3). However, it was difficult to distinguish between possible NOE-peak and noise after the hydrolysis. NOE is typically observed when the distance between two interacting nuclei is less than 6 Å. Hence, these observations suggest that after the hydrolysis the distance between both fluorocarbons increased. In case PERFECTA was dissolved in PFCE within the fractal structure of intact particles, the separation of both fluorocarbons after hydrolysis would be thermodynamically not favored. Thus, this behavior indicates that in intact NPs, PERFECTA is not completely dissolved in PFCE, but both molecules are still associated with each other to produce detectable NOE.

Overall the magnetic resonance properties of PERFECTA/PFCE-PLGA-NPs changed after hydrolysis. A simulation of complex *in vivo* conditions is difficult within an NMR tube. Therefore, here we used a simple method to study the magnetic properties of NPs after the degradation. The complexity of *in vivo* environment, such as physiological fluids and tissue, interactions with different biomolecules, as well as differences in oxygen content can affect the MR properties of fluorocarbons *in vivo*. Nevertheless, the change of the magnetic resonance properties provides the proof of our concept showing that the PERFECTA/PFCE-system is promising for tracking not just biodistribution but also degradation of NPs *in vivo*. Especially, we expect that the different structure of the two used fluorocarbons and the fact that PFCE is a liquid and PERFECTA a solid at physiological temperature, should lead to differences in clearance route of both fluorocarbons after degradation, resulting in different biodistribution. Hence, we expect that combining ^19^F MRI and relaxation time measurements could provide information on degradation of NPs and the environment of fluorocarbons, and thus holds potential to be used to monitor the biodistribution and degradation of drug carriers in later studies.

### 3.4 PERFECTA/PFCE-PLGA NPs are suitable as ^19^F MR imaging agents

The main intended application of these NPs is *in vivo* imaging agents for by ^19^F MRI. Therefore, we investigated their stability in biological milieus, performed cytotoxicity tests and ^19^F MRI.

**Fig. 4:**
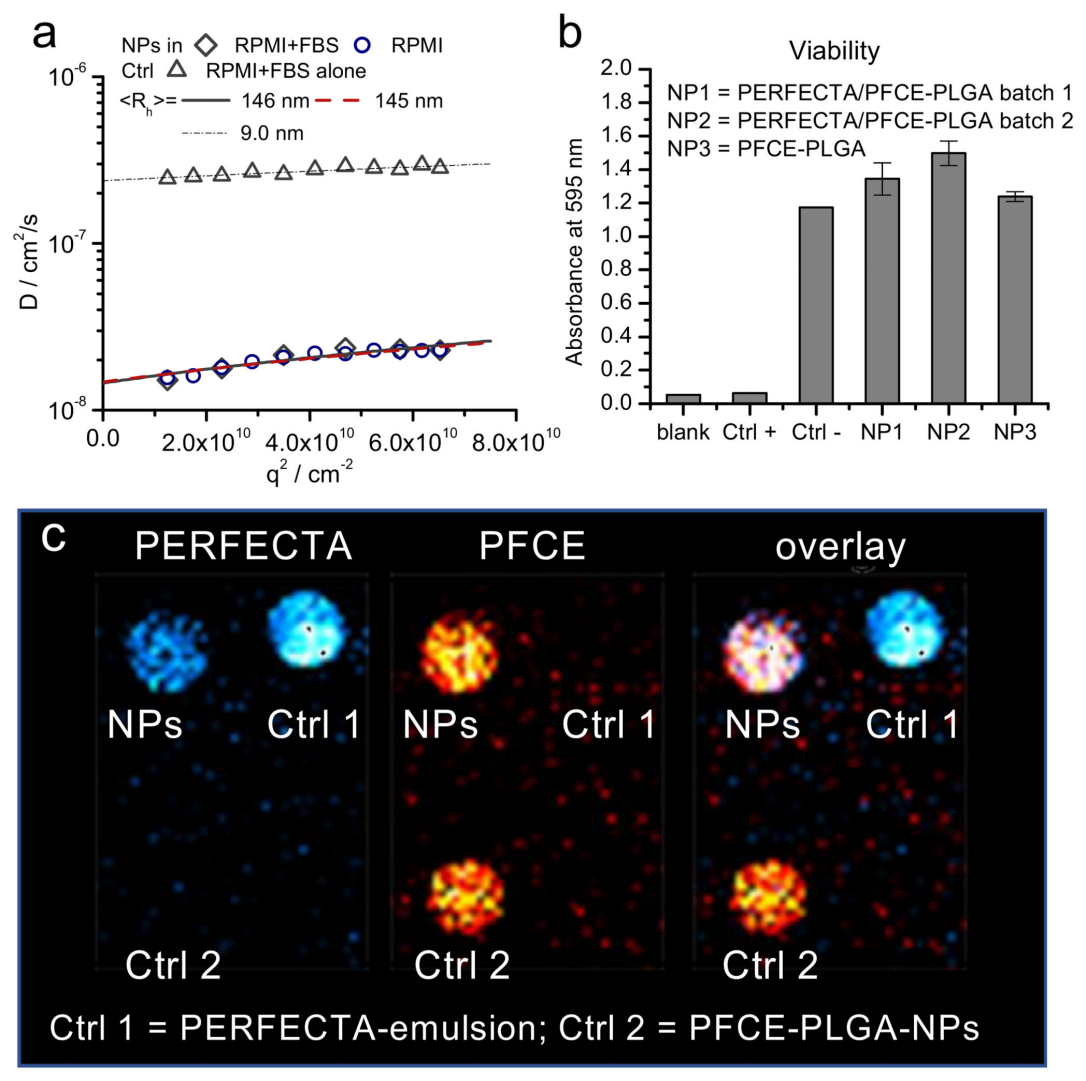
(a) Characterization of NPs in serum-containing cell culture medium RPMI 1640 using multi-angle light scattering. The angular dependency of diffusion coefficient *D* is shown, *q* = scattering vector that is related to scattering angle. The inverse z-average of radii were calculated using Stockes-Einstein equation from *D* at q = 0° that was obtained from linear extrapolation *q* → 0. No changes in size of NPs could be detected in serum-containing medium compared to serum-free medium. c(NP) = 0.01 mg mL^−1^. The measurements were done in triplicate. (b) PERFECTA/PFCE-PLGA-NPs do not affect cell viability, 1×10^6^ cells were incubated in the presence of 2 mg of particles for 72 h (for each condition), followed by the viability analysis with MTT assay. NP1 and NP2 are two different batches of PERFECTA/PFCE-PLGA NPs. Each batch of NPs was tested as a triplicate (c) ^19^F MRI image of PERFECTA/PFCE-PLGA NPs (upper row, left, c = 10 mg/mL) with PERFECTA-emulsion (crtl 1; upper row, right, same PERFECTA-loading as NPs) and PFCE-PLGA-NPs (ctrl 2, lower row, 10 mg/mL). Left: selective excitation of PERFECTA (cyan), middle: selective excitation of PFCE, right: composite. RARE-sequence, transmission bandwidth 1 kHz, receiver bandwidth 15 kHz.

To be used *in vivo*, NPs need to be colloidally stable in physiological milieu. Therefore, we measured multi-angle DLS between 50° and 150° in cell culture medium with and without fetal bovine serum (FBS, Fig. 4a, for different scale compare also Fig. S6). For data analysis in serum, we used a multicomponent analysis developed by Rausch *et al.* to separate the scattering background of serum proteins from the scattering of the particles [35]. The angular dependency of resulting diffusion coefficients of NPs in serum-free and serum-containing medium, and of serum in medium without NPs is shown in Fig. 4a. The hydrodynamic radii, which were obtained from extrapolating q → 0, were 145 ± 7 nm in serum-free medium and 146 ± 16 nm in serum-containing medium. Thus, the NPs were colloidally stable and did not agglomerate in presence of serum, indicating only minor adsorption of biomolecules. As NPs are stabilized with PVA as a non-ionic surfactant, we expect that PVA provides sterical stabilization of nanomaterials and reduces the protein corona formation, as already described for PVA-stabilized colloids in the literature [46].

To investigate the NP effect on cell viability, dendritic cells (DCs) were incubated for 72 h with PFCE/PERFECTA-PLGA-NPs and PFCE-PLGA-NPs at a concentration usually used for cell labeling and then tested by MTT assay. PFCE-PLGA-NPs have now been approved for a clinical trial (clinicaltrials.gov ID NCT02574377) [11,28,29]. There was no significant difference on cell viability between PFCE-PLGA-NPs and PERFECTA/PFCE-PLGA-NPs (Fig. 4b) showing that both types of NPs are not cytotoxic. Thus, we expect that PERFECTA/PFCE-PLGA-NPs also should not display any *in vivo* toxicity and be suitable for biomedical applications.

Finally, we performed ^19^F MR imaging of the NP dispersions (Fig. 4c). As a control we used PFCE-PLGA-NPs and PERFECTA emulsion [23] to show a reference with selective imaging of only one of the molecules. Both PFCE and PERFECTA contain high amount of magnetically identical fluorine atoms and therefore, show singlet signals in their ^19^F NMR spectra. Importantly, the signals of both fluorocarbons are separated by 20 ppm. This large difference in the resonance frequency between the two molecules can be used for selective excitation. Signals from the controls, which were placed in the same imaging phantom, confirmed that we were detecting only one of the fluorocarbons (Fig. 4c, left panel imaging only of PERFECTA, middle only PFCE, right overlay of both fluorocarbons). As we used a conventional sequence for imaging liquid fluorocarbon emulsions (RARE sequence), we expect it should be possible to image the PERFECTA/PFCE-PLGA-NPs *in vivo* with signal-to-noise ratios comparable to PFCE-PLGA-NPs and a concentration limit of 1 mg/mL.

## 4 Conclusion and outlook

^19^F MRI is a powerful technique in the development of nanomedicine. However, ^19^F MRI probes are used just to monitor biodistribution of ^19^F label. Monitoring both biodistribution and degradation of nanoparticles have not been realized thus far. Our goal was to show that the encapsulation of two fluorocarbons within the same particle could be used for imaging of biodistribution and degradation of polymeric drug carriers. Therefore, we developed a fractal nanoscale “two color” MRI probe, loaded with PFCE and PERFECTA. As understanding the colloidal properties is essential for later application of this material, we performed extensive characterization with the emphasis on the internal structure. With 2D ^19^F NMR and SANS we showed that these NPs have a fractal structure and not a core-shell structure, which is typical for fluorocarbon-loaded colloids [8,10,26]. Thus, our particle can be described as an assembly of spherical building blocks, each of them containing PFCE, PERFECTA, and PLGA. Furthermore, we detected ^19^F-^19^F NOE between PFCE and PERFECTA, indicating their close proximity. After the hydrolysis of NPs, we observed changes in ^19^F relaxation times of both fluorocarbons. Moreover, we could not detect a sufficient NOE anymore, indicating that the distance between the two fluorocarbons had increased. These results provide the proof of our concept that both fluorocarbons can separate from each other after a breakdown of NPs. PFCE and PERFECTA both show single magnetic resonances and could be imaged separately using a selective excitation with imaging parameters suitable for *in vivo* studies. Given this property, imaging the biodistribution of PFCE and PERFECTA with different colors combined with relaxation time measurements may enable tracking both biodistribution and degradation of PLGA NPs *in vivo*.

PLGA used in our study is one of the best-established polymers for synthetic therapeutic NPs for clinical and preclinical use [34]. We expect, that NPs described in this work can be tailored to various applications of PLGA or other polymeric NPs, enabling imaging of both biodistribution and degradation of particles. Various techniques for multispectral ^19^F MRI were developed in the recent years [14,47]. Therefore, we expect that this work could have implications for *in vivo* biodistribution and drug delivery and release studies in the future.

## Supplementary Material

Supplementary data to this article can be found online at https://doi.org/10.1016/j.jcis.2019.12.083.

Supplementary Information

## Figures and Tables

**Table 1:** Simultaneous fitting of SANS patterns of PERFECTA/PFCE-PLGA NPs in D_2_O and H_2_O/D_2_O 36:64 (*v:v*) using a fractal sphere model.^i.^

Parameter	D_2_O	H_2_O/D_2_O (36/64)
Vol. fraction	0.0072	0.0072
Radius of fractal block (nm)	4.1	4.1
Polydispersity of fractal block	0.36	0.30
Fractal dimension	3.05	3.05
Correlation length (nm)	41	41
SLD sphere (Å^−2^)	4.74 × 10^−6^	3.15 × 10^−6^
SLD solvent (Å^−2^)	6.36 ×10^−6^	3.87 ×10^−6^
Bkg	0.011	0.023
Chi^2^/Npts	3.5	
R_g_ of NPs (nm)	102	

i Volume fraction and SLD of solvent were fixed; block radius, fractal dimension and correlation length were fitted simultaneously.

**Table 2:** Relaxation times of PERFECTA/PFCE NPs before and after hydrolysis compared to PFCE NPs.^i^

NPs	PFC	T_1_ [ms]	T_2_ [ms]
PERFECTA/PFCE-PLGA-NPs	PERFECTA	542	145
	PFCE	620	273
PFCE-PLGA-NPs NPs after hydrolysis	PFCE	794	492
PERFECTA/PFCE-PLGA	PERFECTA	520	207
	PFCE	684	372
PFCE-PLGA	PFCE	775	339

i NPs in D_2_O, 471 MHz.
