# Supplementary material for: Nanoparticles for “two color” 19F magnetic resonance imaging: Towards combined imaging of biodistribution and degradation
Source: J Colloid Interface Sci. Author manuscript; Available in PMC 2020 Mar 5. (PMC7058420; doi:10.1016/j.jcis.2019.12.083)
Supplement: Supplementary Information [file EMS85882-supplement-Supplementary_Information.pdf]

Supporting information to

"Two Color" Nanoparticles for ^{19}F Magnetic Resonance Imaging: Towards Combined Imaging of Biodistribution and Degradation

Olga Koshkina,[1,¶,*] Paul B. White,[2,‡] Alexander H. J. Staal,[1,‡] Ralf Schweins,[3,‡] Edyta Swider,[1] Ilaria Tirotta,[4] Paul Tinnemans,[2] Remco Fokkink,[5] Andor Veltien,[6] N. Koen van Riessen,[1] Ernst R. H. van Eck,[2] Arend Heerschap,[6] Pierangelo Metrangolo,[4] Francesca Baldelli Bombelli,[4] Mangala Srinivas[1,*]

[1]Department of Tumor Immunology, Radboud Institute for Molecular Life Sciences, Radboud University Medical Center, Geert Grooteplein Zuid 26/28, 6525 GA Nijmegen, The Netherlands

[2]Institute for Molecules and Materials, Radboud University, Heyendaalseweg 135, 6525 AJ Nijmegen, The Netherlands

[3]Institut Laue – Langevin, DS / LSS, 71 Avenue des Martyrs, Grenoble

[4]Laboratory of Supramolecular and Bio-Nanomaterials (SupraBioNanoLab), Department of Chemistry, Materials, and Chemical Engineering "Giulio Natta", Politecnico di Milano, Via Luigi Mancinelli 7, 20131 Milan, Italy

[5]Department of Agrotechnology and Food Sciences, Physical Chemistry and Soft Matter, Wageningen University, 6708 WE Wageningen, Netherlands

[6]Department of Radiology and Nuclear Medicine, Radboud University Medical Center, Geert Grooteplein Zuid 10, 6525 GA Nijmegen, the Netherlands

* Corresponding Author:

mangala.srinivas@radboudumc.nl

koshkina@mpip-mainz.mpg.de

‡ Authors contributed equally

¶Present address OK: Max Planck Institute for Polymer Research, Ackermannweg 10, 55128 Mainz, Germany

1. Synthesis of nanoparticles loaded only with PERFECTA

1.1. Synthesis

Poly(vinyl alcohol) (PVA, 500 mg) was dissolved in ultrapure water (25 g) in a 50 mL round-bottom flask. PERFECTA (100 mg) was dissolved in 2.5 mL solkane® and quickly mixed with PLGA (100 mg) in 1 mL of a mixture of dichloromethane / ethyl acetate 1:1(*v:v*). The resulting suspension was added quickly to PVA through a glass pipette, while the sonication was started. The addition of PERFECTA has to be done rapidly, as the PLGA and/or PERFECTA begin to precipitate right after mixing. The sonication was carried out in ice-water bath using a branson digital sonicator at 40% amplitude equipped with a 3.2 mm microtip. Afterwards the resulting emulsion was stirred over night at a room temperature to evaporate the solvent. The particles were isolated by centrifugation (35 min at 16000 g), washed three times with ultrapure water and freeze-dried from water. The yield of the batch used for solid state NMR was 87 mg, the encapsulation of PERFECTA from NMR (internal TFA reference in D_2O) 13 wt.-% and the hydrodynamic radius 215 nm (PDI = 0.2, Malvern Zeta sizer).

1.2. Summary of additionally tested conditions

The above-described process was generally reproducible in terms of PERFECTA-encapsulation and size; however, some batch-to-batch variation of yield was observed.
As mentioned in the main text, the preparation of the PERFECTA-only nanoparticles turned out to be difficult. The following Table S1 summarizes **non-working** conditions, at which some particles were isolated, but either size or PERFECTA encapsulation were not sufficient. When poly(vinyl pyrrolidone) or Tween 20 were used as surfactant instead of PVA no particles were isolated and aggregation was visible shortly after sonication.

Table S1. Further tested synthesis conditions for synthesis of PERFECTA-PLGA-nanoparticles without PFCE (n.d. = not determined).

$m_{PERFECTA}$ (mg)	solvent	Other additives	sonication	R_h (nm)	PDI	PERFECTA in isolated NPs (wt.-%)
102.6	3 mL HFIP	-	3 min, 40%	103	0.14	3
203.5	3 mL HFIP	-	3 min, 40%	n.d.		0

201.4	1 mL HFIP, 2 mL DCM	-	3 min, 40%	151	0.23	2
101	3 mL HFIP	30 mg lecitin	2 min RT[i], 2 min ice	n.d.		0
101	0.5 mL HFIP, 2.5 mL solkane	-	3 min, 40%	403	0.31	1
101	1 mL solkane 2 mL AcOEt	-		340	0.28	0
97	2.5 mL solkane, 0.5 mL AcOEt	-	3 min, 40%	1320	0.45	28
50	1 mL DCM, 2 mL solkane	20 mg lecitin	3 min, 40%	n.d.		0

[i]RT = room temperature; the sonication at room temperature was interrupted for ca. 1 min twice due to foam formation.

2. Additional characterization data

2.1. Multiangle Dynamic and Static Light Scattering (DLS, SLS) of PERFECTA/PFCE-PLGA NPs

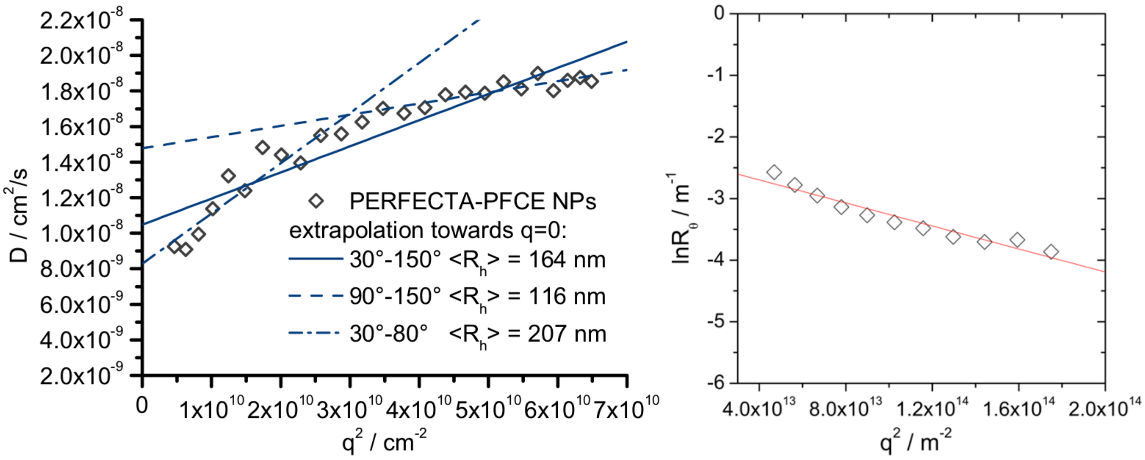

Figure S1. Left: Multi-angle DLS of PERFECTA/PFCE-PLGA NPs: angular dependency of apparent diffusion coefficient D plotted versus the squared scattering vector q. The inverse z-average of hydrodynamic radius obtained from extrapolation q→0 was $\langle R_h \rangle_{1/z}$ = 164 nm. When only apparent diffusion coefficient value at small angles are used to calculate the hydrodynamic radius, an R_h = 116 nm is obtained which is closer to cryoSEM radius. At smaller angles (between 30° and 80°) a larger radius of 207±16 nm resulted. c_{NP} = 0.01 mg/mL. Right: Static light scattering of PERFECTA/PFCE-PLGA NPs. Guinier-plot demonstrated the radius of gyration R_g = 167 nm. C(NP) = 0.001 mg/mL

2.2. Solid state NMR spectroscopy

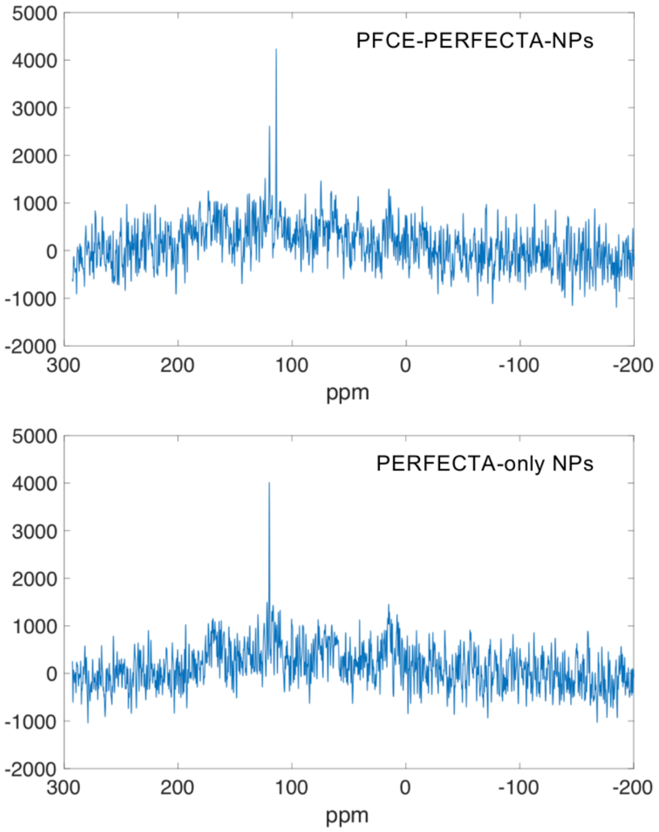

Figure S2. Characterization of PERFECTA/PFCE-PLGA-NPs and PERFECTA-PLGA NPs by ^{13}C solid state NMR spectroscopy.

2.3. Small Angle Neutron Scattering

Table S2. Coherent neutron scattering length densities (SLD) of nanoparticle components

Compound	Molecular Formula	Bulk density (g/cm-3)	SLD (Å^{-2})
PLGA[1]	$C_5H_6O_4$	1.34	2.11×10^{-6}
PFCE[2]	$C_{10}F_{20}O$	1.78	3.87×10^{-6}
PERFECTA	$C_{21}H_8F_{36}O_4$	2.09	4.20×10^{-6}
heavy water	D_2O	1.1	6.36×10^{-6}
light water	H_2O	1.0	-0.59×10^{-6}
H_2O/D_2O (36/64 *v:v*)	x_{H2O} = volume fraction of H_2O<br>$SLD_{solvent} = x_{H2O}\times SLD_{H2O}+(1-x_{H2O})\times SLD_{D2O}$		3.87×10^{-6}
H_2O/D_2O (61/39 *v:v*)			2.11×10^{-6}

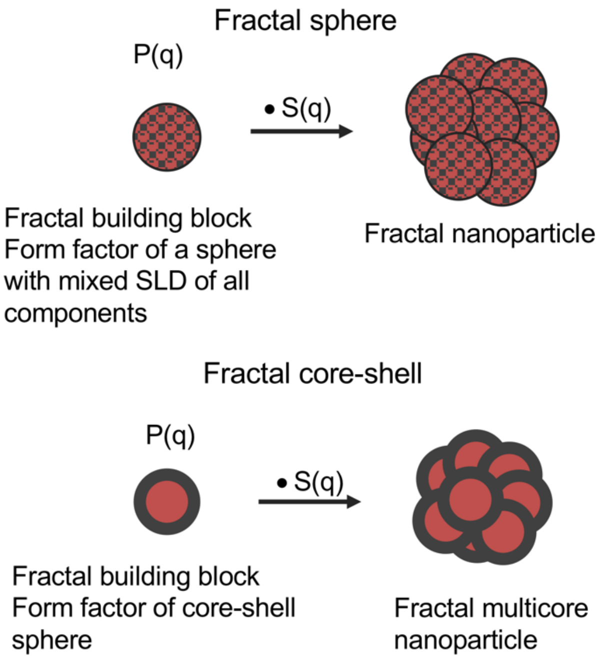

Figure S3. Schematic of Teixeira's fractal sphere model (NIST model FractalPolySPhere), which was used for PERFECTA/PFCE-PLGA NPs compared to fractal core-shell model (NIST model FractalPolyCore), which was previously applied to fit SANS patterns of PFCE.

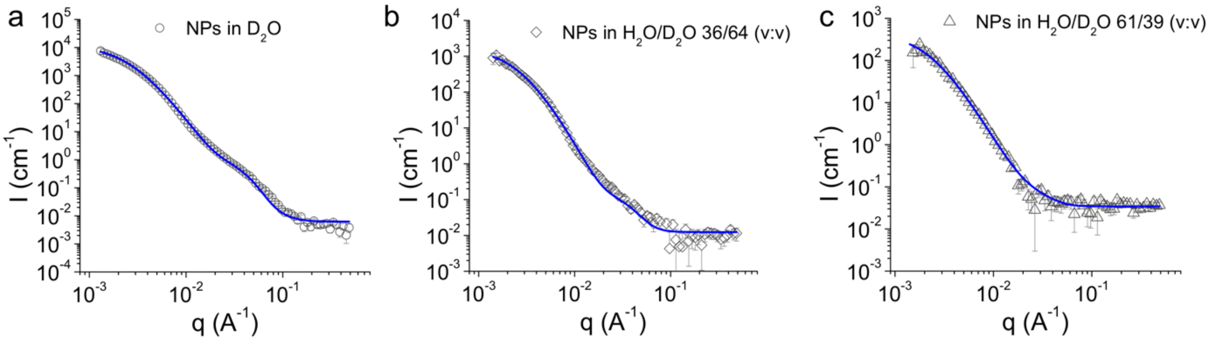

Figure S4. SANS scattering patterns of PERFECTA/PFCE-PLGA NPs measured at the ISIS beamline. A) Nanoparticles in D2O b) nanoparticles in H_2O/D_2O 36/64 (*v:v*; PFCE is matched) c) nanoparticles in H_2O/D_2O 61/39 (*v:v*; PLGA is matched). c(NP) =10 mg/mL-

Table S3. Fitted and calculated parameters obtained using a fractal sphere model for fitting SANS patterns of PERFECTA/PFCE-PLGA NPs in different solvents measured at ISIS neutron source.

Parameter	D_2O	H_2O/D_2O 36/64	H_2O/D_2O 61/39
Vol. fraction	0.0072	0.0072	0.0072
Radius of fractal block (nm)	4.1	4.1	4.0
Polydispersity of fractal block	0.35	0.36	0.36
Fractal dimension	3.08	3.13	2.95
Correlation length (nm)	40	38	43
SLD sphere (Å^{-2})	4.85×10^{-6}	3.31×10^{-6}	2.44×10^{-6}
SLD solvent (Å^{-2})	6.36×10^{-6}	3.87×10^{-6}	2.11×10^{-6}
Background	0.006	0.012	0.03
Chi^2/Npts	7.8	2.7	1.0
R_g of nanoparticles (nm)	100	97	104

Methods for measurements at the ISIS neutron source (sans2d)

SANS measurements were repeated at the SANS2D small-angle diffractometer at the ISIS Pulsed Neutron Source (STFC Rutherford Appleton Laboratory, Didcot, U.K.; http://www.isis.stfc.ac.uk).[3] Here, a collimation length of 12 m and incident wavelength range of 1.75 – 12.5 Å was employed. Data were measured simultaneously on two 1 m^2 detectors to give a q-range of 0.0015 – 0.85 $Å^{-1}$. The small-angle detector was position 12 m from the sample and offset vertically 80 mm and sideways 100 mm. The wide-angle detector was position 5m from the sample, offset sideways by 860 mm and rotated to face the sample. The magnitude of the scattering wave vector q is defined as:

$$Q = \frac{4\pi \sin\frac{\theta}{2}}{\lambda}$$

where θ is the scattered angle and λ is the incident neutron wavelength. The beam diameter was 8 mm. Each raw scattering data set was corrected for the detector efficiencies, sample transmission and background scattering and converted to scattering cross-section data ($\partial\Sigma/\partial\Omega$ vs. q) using the instrument-specific software (http://www.mantidproject.org). These data were placed on an absolute scale (cm^{-1}) using the scattering from a standard sample (a solid blend of hydrogenous and perdeuterated polystyrene) in accordance with established procedures.[4]
Samples in D_2O and H_2O/D_2O 36/64 (*v:v*) were measured in 2 mm quartz cuvettes, while for samples in H_2O/D_2O 61/39 (*v:v*) 1mm cuvettes were used. In total five different batches of nanoparticles with and without gadoteridol and two different batches were measured. Measurement data that was not included in this manuscript is available from the authors.
Data analysis was done using the NIST SANS Macro for Igor Pro (Wavemetrics).[5]

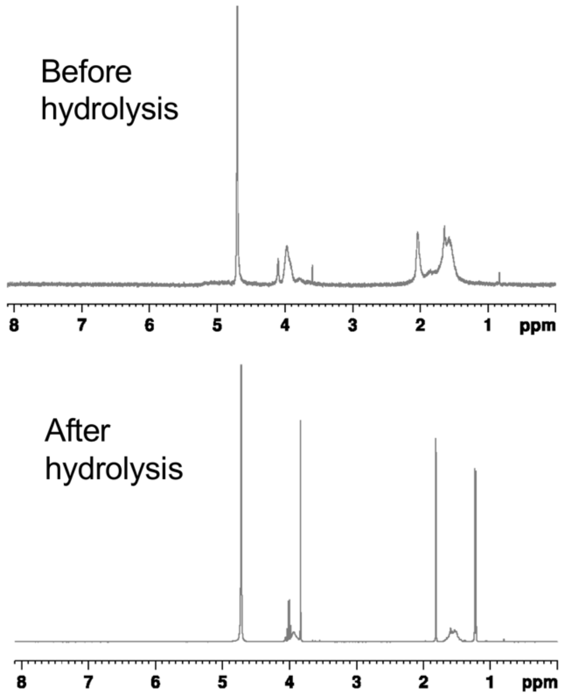

Figure S5. **^{1}H NMR spectroscopy of PERFECTA/PFCE-PLGA NPs before (upper image) and after (lower image) hydrolysis. In intact nanoparticles only signals from PVA can be detected, while PLGA is not visible due to low mobility of polymeric chains that results in short transverse relaxation time. After hydrolysis signals of lactic and glycolic acid can be detected with ^{1}H NMR spectroscopy. D_2O, 400 MHz.**

2.4. DLS of PERFECTA/PFCE-PLGA NPs in serum

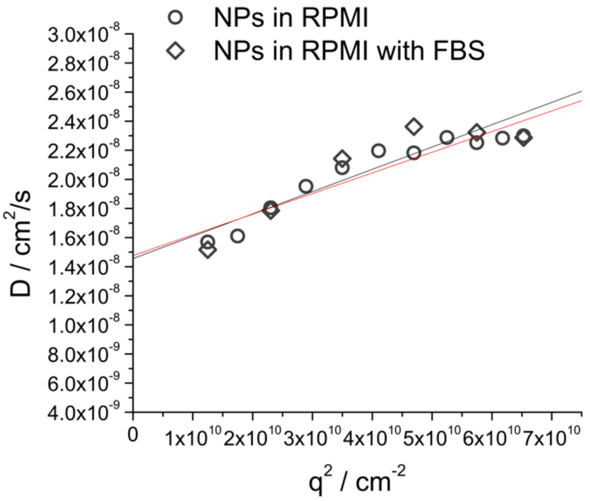

Figure S6. Characterization of PERFECTA/PFCE-PLGA NPs under physiological conditions-zoom-in on the angular dependency of diffusion coefficients of nanoparticles (compare fig.2 b in the main text).

1. B. Yang, J. P. Lowe, R. Schweins and K. J. Edler, *Biomacromolecules*, 2015, **16**, 457-464.
2. http://www.exfluor.com/msds.php?id=F15-crown-5).
3. R. K. Heenan, S. E. Rogers, D. Turner, A. E. Terry, J. Treadgold and S. M. King, *Neutron News*, 2011, **22**, 19-21.
4. G. D. Wignall and F. S. Bates, *J. Appl. Crystallogr.*, 1987, **20**, 28-40.
5. S. R. Kline, *J. Appl. Crystallogr.*, 2006, **39**, 895-900.
